# Evaluating an emotion coaching programme for parents of young adolescents attending Child Adolescent Mental Health Services (CAMHS) in New Zealand: protocol for a multi-site feasibility trial including co-design with service users

**DOI:** 10.1186/s40814-023-01282-6

**Published:** 2023-04-27

**Authors:** Zara Mansoor, James Stanley, Sarah Fortune, Sophie Havighurst, Elliot Bell

**Affiliations:** 1 Department of Psychological Medicine, University of Otago, PO Box 7343, Wellington, 6242 New Zealand; 2 Biostatistics Group, University of Otago, Wellington, New Zealand; 3 School of Population Health, The University of Auckland, Auckland, New Zealand; 4 Department of Psychiatry, Mindful Centre for Training and Research in Developmental Health, The University of Melbourne, Melbourne, Australia; 5 Department of Psychological Medicine and Rehabilitation Teaching and Research Unit, University of Otago Wellington, Wellington, New Zealand

**Keywords:** Anxiety, Depression, Young people, Adolescents, Parenting, Families, Mental health, Psychological intervention, Feasibility trial

## Abstract

**Background:**

Early adolescence is a time of increased vulnerability for the development of common mental health conditions such as anxiety and depression (internalising outcomes). Current treatments such as cognitive-behavioural therapy and antidepressant medication are focused on the individual and have small effect sizes, particularly in real-world clinical settings such as the public Child Adolescent Mental Health Services (CAMHS). Parents are an important and under-utilised resource in treating these conditions in young adolescents. Teaching parents how to respond to their young person’s emotions can improve emotion regulation and reduce internalising outcomes. One emotion-focused programme for parents of this age group is Tuning in to Teens (TINT). This is a structured, manualised skills group for parents only focused on teaching skills to coach young people through their emotional experiences. This study aims to investigate the impact of TINT in the clinical setting of publicly funded CAMHS in New Zealand.

**Methods:**

The trial will evaluate the feasibility of a two-arm multi-site randomised control trial (RCT). Participants will be 10–14-year-olds referred to CAMHS in Wellington, New Zealand, with anxiety or depression, and their parents or guardians. Arm 1 will be parents attending and implementing TINT (in addition to the usual care received at CAMHS). Arm 2 will be usual care only. TINT groups will be facilitated by CAMHS clinicians who have been trained in the programme and will be delivered over 8 weekly sessions. Prior to the RCT, a co-design methodology will be used with service users to inform outcome measures used in the trial. A group of service users meeting the RCT criteria will be recruited to take part in workshops to help determine their priority outcomes. Measures based on the results of workshops will be included in the outcome measures. The primary feasibility outcomes will be the recruitment and retention of participants, acceptability of the intervention for service users and clinicians and acceptability of outcome measures.

**Discussion:**

There is a need to improve outcomes for the treatment of adolescent anxiety and depression. TINT is a programme with the potential to enhance outcomes for those accessing mental health services by providing targeted support to parents of adolescents. This trial will inform whether a full RCT is feasible to evaluate TINT. Including service users in the design will increase its relevance of an evaluation in this setting.

**Trial registration:**

The Australian New Zealand Clinical Trials Registry (ACTRN): ACTRN12622000483752. Registered on 28 March 2022.

**Supplementary Information:**

The online version contains supplementary material available at 10.1186/s40814-023-01282-6.

## Background

The transition from childhood to adolescence is a time of change and challenge. One in four adolescents will experience a common mental health condition such as anxiety or depression, with these conditions accounting for 16% of the global burden of disease in this age group [1, 2]. Early adolescence is a particularly vulnerable period, with half of all mental health conditions in adolescence developing by age 14 years [2, 3]. Longitudinal studies have consistently found mental health difficulties in childhood, and adolescence predicts poorer mental health and wellbeing in adulthood [4, 5].

In New Zealand, Child and Adolescent Mental Health Services (CAMHS) provide publicly funded care for adolescents with moderate to severe mental health difficulties. These services face increasing demand, with rising rates of mental health difficulties in young New Zealanders, particularly for Māori, the indigenous population of New Zealand [6]. As the prevalence of mental health conditions has risen, referrals to CAMHS have also increased, particularly for the 10–14 year age group [7].

Family systems theory describes the importance of the family unit as a whole in child development. Parents play a crucial role in shaping a child’s health and wellbeing through their relationships and family dynamics. Consistent with this theory, a variety of parenting factors are significantly related to adolescent depression and anxiety [8]. With various barriers to accessing mental health support for young people, families are also likely to be the first (and often only) source of support available [9]. Despite the rationale for including parents as treatment targets for this population, treatments are often individually focused with the first-line treatment for adolescent anxiety and depression typically Cognitive Behaviour Therapy (CBT) and/or antidepressant medication [10].

While there is a reasonable body of evidence for the effectiveness of CBT in younger populations, effect sizes are often small and a systematic review of CBT for youth anxiety has found this may be negligible when compared to active control conditions such as guided self-help [11]. There is also evidence that effect sizes for CBT and other talking therapies are smaller for adolescent versus adult populations [12]. A recent review and meta-analysis of antidepressant medication in children and adolescents also found effect sizes to be small [13]. The effectiveness of individually focused treatments is further reduced when caring for adolescents with substantial mental health needs in settings like CAMHS, where family and other systematic factors frequently contribute to the complexity of their challenges [10].

In New Zealand, there is an obligation under Te Tiriti ō Waitangi (The Treaty Of Waitangi) to bicultural practice that meets the needs of indigenous Māori. Models of Māori wellbeing such as ‘Te Whare Tapa Wha’ (The House with Four Sides) include whānau (roughly translated as family, however, encompassing a wider meaning of social networks) as a key pillar of health [14]. Treatment options that include parents and caregivers are important to align with this perspective. Consistent with this, a survey of Māori accessing CAMHS found family involvement a key variable in satisfaction with care [15]. This is also likely to be relevant for other priority ethnic groups in New Zealand including Pacific peoples [16].

Parent interventions can be broadly divided into behaviour or emotion-focused interventions. Behaviour-focused interventions typically focus on modifying child behaviour through the use of rewards, punishments, structured routines and clear expectations. Emotion-focused parent interventions focus on improving parent-child relationships and emotion regulation. An emotion-focused approach may be particularly indicated for an older age group and where internalising problems are the focus. A review of emotion-focused parenting interventions found positive impacts on a range of parent and child-related outcomes, including emotion regulation [17]. This review did not include meta-analysis and the included studies generally covered programmes for younger age groups with less evidence for adolescents.

*Tuning in to Teens*^*TM*^ (TINT) is an emotion-focused intervention for parents tailored specifically to this age group. This programme has been adapted from a programme for younger children, *Tuning in to Kids*^*TM*^*,* developed by a team at the University of Melbourne [18]. These are evidence-based programmes based on research into emotion socialisation, the processes of learning about emotions and how parents can promote their children’s emotional intelligence [13]. TINT aims to support parents to better ‘tune in’ to the emotions of their children by becoming ‘emotion coaches’. Emotion coaching is defined as an adaptive emotion socialisation style which encourages understanding and acceptance of emotional experience [19]. This style of responding is associated with better mental wellbeing, along with other positive outcomes such as improved social skills and academic performance [19, 20]. In contrast to emotion coaching, other styles of responding to emotions such as permissive, dismissive, or disapproving responses are linked to poorer mental health [8]. The TINT programme also includes information on typical adolescent development and other strategies for regulating emotion such as relaxation and mindfulness techniques.

There is evidence for the benefit of TINT in the 10–14 age group in a community setting from a randomised control trial in Melbourne, Australia [19]. Participants were young adolescents transitioning to high school (*n*=224, mean age=12, 49% boys) and their primary caregivers, who were randomised by the school to either receive TINT or not. Caregivers who completed the programme had significant improvements in their own emotion regulation skills, a change in their parenting responses towards increased emotion coaching and these changes were associated with reduced internalising difficulties in their young adolescents [19, 21].

In New Zealand, several CAMHS have been implementing TINT with positive anecdotal feedback, however, the programme has not been formally evaluated. As this will be the first study to evaluate TINT in CAMHS in New Zealand, a feasibility trial has been chosen to assess the various research challenges in this real-world setting before embarking on a larger-scale trial. While randomised experimental designs are gold-standard for testing effectiveness in health settings, testing feasibility factors on a smaller scale before proceeding to larger and more resource-intensive trials is recommended when interventions are complex and interact with other factors [22]. There is also evidence from a previous RCT looking at a manualised approach to delivering CBT across several CAMHS in New Zealand that recruitment rates were lower than expected, and there was difficulty recruiting representative numbers of Māori and other priority ethnicity groups [23]. A national inquiry into Mental Health in 2018 reported a 70% increase in demand for CAMHS between 2006 and 2016, while at the same time noting significant workforce shortages across the field [24]. These continue to be critical issues in the field that may impact research feasibility.

As the overall aim of an evaluation is to determine treatment effectiveness in a specific setting, there is also a question of how outcomes should be measured. In health research, service-user and research or clinician agendas may not align [25]. For instance, drug trials are given preference by researchers whilst non-drug treatments are preferred by service users [18, 19]. In youth mental health, as outcomes are often focused on individual symptoms, these may not reflect other mechanisms of systemic change or what is meaningful for young people and families [26]. To address this, the current study will include a novel co-design methodology working with parents and young adolescents to input into the measures used in the trial. This is a unique and contemporary approach intended to maximise the relevance of findings to service users in this complex real-world setting. Service users will be asked about their priority outcomes for a programme like TINT so that this can be incorporated into the programme evaluation.

### Aims

The primary aim of this study is to examine the feasibility of a randomised control trial (RCT) of an intervention for parents, *Tuning in to Teens* ^TM^ (TINT), delivered in addition to usual care for young adolescents aged 10–14 years who have presented to mental health services with a primary difficulty related to anxiety or depression. The feasibility study will aim to identify and address issues that may arise in the current CAMHS context in New Zealand. The specific objectives will include the assessment of factors related to the recruitment and retention of participants, acceptability and feasibility of the intervention and acceptability and feasibility of outcome measures. A secondary aim is to include service users in the development and decision making around how to measure the impact of TINT in CAMHS. The aim of this is to incorporate a measure that will capture specific service user needs in our community so this can be included in decision making around the benefit of this intervention.

The results of this feasibility study will inform the future development of a full-scale RCT to test the hypothesis that this additional parent-focused intervention will improve outcomes for young adolescents with internalising difficulties in a clinical child and adolescent mental health setting. Testing the feasibility of the study design will ensure that any larger-scale trial is conducted in a manner that is both scientifically rigorous and feasible in this real-world setting. Including a service-user perspective in outcome measures will help ensure relevance for those the intervention is intended to benefit.

## Methods/design

### Study design

This feasibility study has a mixed methods design with two phases:

- Phase I: The first phase of the study will use a co-design methodology to determine how the impact of TINT is measured in the trial. This methodology has been chosen to increase the relevance of the research in line with a broader objective of making sure that we are meeting service user needs in CAMHS. Co-design has proven value in other health settings in New Zealand [26–28]. In co-design, service users are viewed as experts alongside clinicians and researchers who work together to integrate their experiences. A group of 10–14-year-olds engaged with CAMHS in the Wellington region, and their parents will be recruited to participate in a co-design workshop. Workshops will be facilitated by the research team with the aim of co-designing an outcome measure to be included in an evaluation of TINT. The co-design process will be guided by a multi-stage approach incorporated in other New Zealand health research projects: engage, plan, explore, develop, decide and change [29]. Following the exploration and development of themes in the workshops, a measure will be drafted for each group (parents and 10–14-year-olds) for inclusion in the trial. This may take the form of an adaption of existing questionnaires or an additional question or scale to be included.
- Phase II: The second and final phase of the study will be a feasibility study using an experimental methodology of a randomised control trial (RCT). There will be two arms to the trial:
-
  1. TINT for parents + usual care in CAMHS.
  2. Usual care in CAMHS only.

The comparison of TINT plus usual care with usual care only (described later) gives a pragmatic and ethical comparator in a real-world clinical setting. This design also gives an accurate representation of practice in CAMHS where different types of interventions are often layered together. If a larger scale trial is feasible, this design will allow for testing of the hypothesis that adding TINT to usual care improves outcomes for young adolescents in CAMHS.

**Fig. 1:**
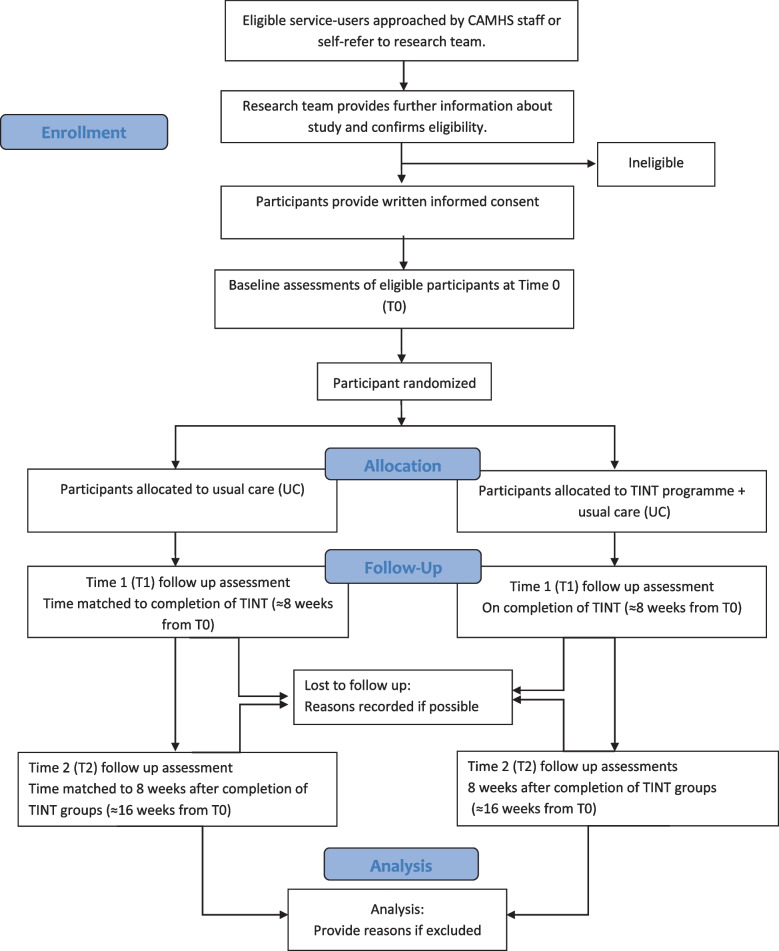
Flow chart of phase II RCT

**Fig. 2:**
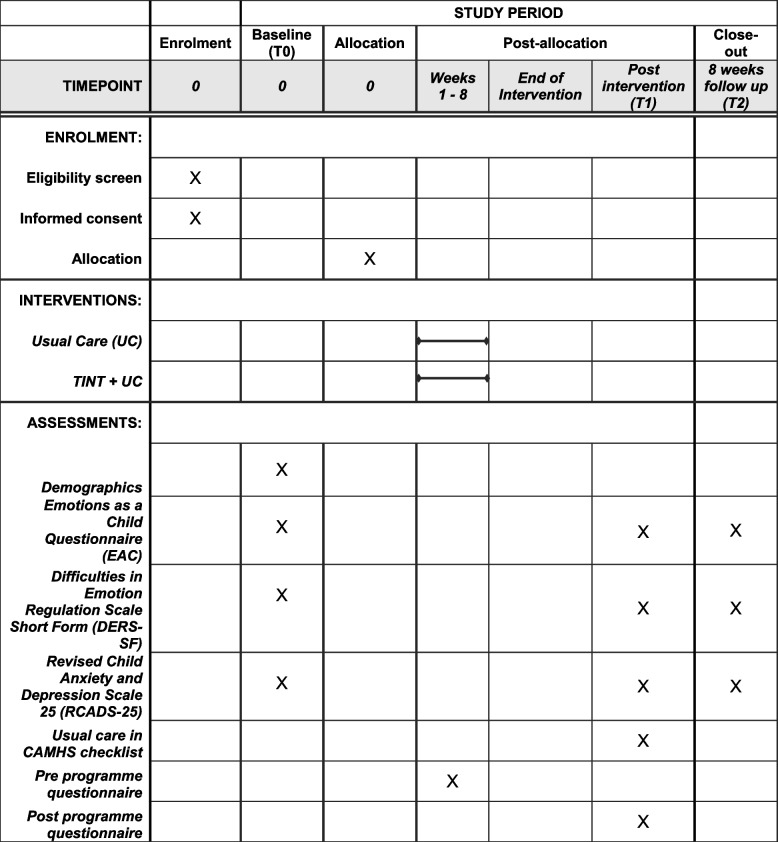
Schedule of enrolment, interventions and assessments for phase II RCT

Participants will be randomly allocated to a study arm. For those allocated to receive TINT in addition to usual care, parents will complete a TINT group programme at CAMHS (described later). TINT groups will be facilitated by at least two CAMHS clinicians and involve eight sessions of 2 h each week. Those allocated to control will be given the option of completing TINT after the study period. Outcome measures will be collected prior to starting groups (T0), immediately after completing TINT/matched for the control arm (T1) and 8 weeks after completing TINT/matched for the control arm (T2). See Fig. 1, the flow chart of phase II RCT. The trial will follow the Standard Protocol Items for Clinical Trials (SPIRIT) statement [30]. See Fig. 2 for the schedule of enrolment, interventions and assessments for phase II RCT and SPIRIT checklist in additional files (Additional file 1: SPIRIT checklist).

### Study setting

The setting for this study is CAMHS services in the Wellington region of New Zealand. New Zealand is a high-income country with a population of around 5 million people of mixed ethnicities. Its history and culture have been affected by European colonisation with ongoing impacts on the indigenous population of Māori. Wellington is the capital city, and the greater Wellington region is home to around 550,000. Based on 2018 census data, the regional population was 74.6% European/Pākehā, 14.3% Māori, 8.4% Pacific peoples, 12.9% Asian and 3.3% other ethnicities [31]. CAMHS form part of the public health service in New Zealand which provides free universal care to eligible citizens, funded by general taxes [32]. Public health services have been administered by 20 regional District Health Boards (DHBs), which as of 2022 are in the process of integration into one national administrative body. In the greater Wellington region, there are five mainstream CAMHS and two speciality services: one for Māori families and one for Pacific peoples. Those from Māori or Pacific communities may choose whether to access mainstream or speciality services.

### Participants

Participants will be young adolescents aged 10–14 years who have been referred to CAMHS with a presenting difficulty related to anxiety or depression, and their parents or guardians (including non-birth parents e.g. step-parents, other family members in a parenting role or legal guardians). Participant criteria will be the same for both phases so that those designing the measure are from the same population intended for the evaluation. Although TINT is a parent-only intervention, 10–14-year-olds will also be asked to provide outcome data on their internalising symptoms and proposed mechanisms of change related to parenting and emotion regulation.

### Inclusion criteria

Inclusion criteria for adolescents are as follows:

• Aged 10 to 14 years at the time of recruitment.
• Accepted into a CAMHS in the Wellington region.
• Primary presenting difficulty related to anxiety (including any DSM-5 Anxiety Disorder) or depression (including any DSM-5 Depressive Disorder).
• Sufficiently competent and fluent in the English language to complete outcome measures.
• Have at least one parent/guardian who meets the study inclusion criteria.

Inclusion criteria for parent(s)/guardian(s) are as follows:

• Aged over 18 years.
• Have a care of a child in CAMHS who meets the inclusion criteria.
• Sufficiently competent and fluent in the English language to complete the intervention group and outcome measures.
• Available and willing to complete the intervention group (determined prior to randomisation).

### Exclusion criteria

• Presenting difficulty is not anxiety/depression.
• Communication difficulty (e.g. lacking fluency in English or having a learning difficulty which impacts the ability to give consent or complete outcome measures).

### Recruitment and target sample size

All participants will be recruited through participating CAMHS. General information about the study will be provided to these services and advertised via printed material displayed in waiting rooms and other public areas of these services. Clinical staff members may also send out individual invitations to current service users who may meet the study criteria. Interested participants will be invited to contact the research team directly.

Recruitment for phase I (co-design) will aim to recruit six to ten parent participants and six to ten 10 to 14-year-olds. Recruitment for phase II, the RCT feasibility study, will aim to recruit 50 participants (25 per arm) as an approximate upper limit: the total numbers recruited in the study period will form part of the feasibility assessment.

As per guidelines for feasibility trials, the sample size for the feasibility trial has been set to address feasibility questions rather than a full-scale RCT and within the constraints of the study budget and time frame [33]. The planned time frame for referral is one quarter of a school year. In the target range across local services, there are approximately 150 total referrals per quarter [34].

There are a recommended number of a maximum of eight to twelve participants per TINT group or approximately five to six families (assuming two family members join If all five possible sites run an intervention group in the study period this sets an upper limit of approximately 30 families per arm or 60 families overall in that quarter. The final sample size will give a reasonable estimate on per-quarter recruitment. For example, if 50 families are recruited, the true per-quarter recruitment rate would fall between 37 and 66 families (95% confidence interval). The calculation of the recruitment rate out of eligible service users will have a precision of ±8% (confidence interval for recruitment rate). This will enable calculations of the time frame needed for a fully powered trial.

### Consent and eligibility

The principal investigator will provide further information to those who express interest in the study, screen them for inclusion/exclusion criteria and obtain informed consent. All potential participants will be provided with written information about the study and provided with options to discuss the study based on their personal preference (phone, face to face, email, text). For those aged 10 to 14, an assessment of their ability to provide informed consent will be made by the principal investigator who has expertise working with young people in mental health settings. This may include obtaining additional information from family members and/or the care team at CAMHS as required, for example, to determine the presence of any learning or cognitive difficulties that may impact the ability to consent. For those requiring support to provide consent, this will be sought from a parent/guardian with assent sought from the young adolescent. Participants may withdraw at any time on their own request without providing a reason. For the first phase, the co-design of measure recruitment will be targeted at both 10–14-year-olds and their parents. For the second phase, the feasibility RCT, recruitment will be targeted at parents as the primary contacts, with their children then asked for consent to complete outcome measures (optional).

### Randomisation, allocation and blinding

Randomisation and allocation apply to phase II (RCT feasibility). A pre-determined allocation sequence will be created by the team statistician (JS) and uploaded to REDCap (Research Electronic Data Capture) trial management software [35]. Allocation will be on a 1:1 basis (i.e. equal size groups for intervention and control arm).

As separate TINT groups will be run in each participating service, separate randomisation schedules will be constructed for each centre to allow allocation on this basis. Randomisation schedules will be constructed using a permuted block randomisation [36] with a mean block length of 4 allocations (2 intervention, 2 control). Participants will be recruited during one school term (approximately 10 weeks) with the intervention run in the following term. This randomisation schedule will allow for accrual over the term and participants to be notified of allocation as they enter the study.

Following confirmation of eligibility and informed consent, participant data will be entered in REDCap which will be used to draw the next allocation from the random allocation sequence for the appropriate service (the allocation schedule is not visible). The research team member responsible for this step will communicate these allocations to relevant staff members to coordinate running the TINT groups. Due to the nature of the intervention, participants and clinicians will not be blind to intervention status; however, the researcher collecting the outcome data will be blinded to allocation when recording outcomes to reduce the risk of bias. The analysis will be conducted blind to outcome status, including leaving analysis of responses regarding the perception of the TINT sessions until after the main analysis is complete and the study subsequently unblinded for reporting.

### Intervention

TINT is designed to be delivered in a group format over weekly sessions of 2 h each. It is a parent-only intervention. An 8-week format will be used for the study as the recommended format for clinical settings. The focus of sessions is teaching emotion coaching skills. Parents are taught key steps to emotion coaching with a focus on awareness, listening and acceptance and then, if necessary, problem-solving or negotiating boundaries with their adolescent.

Sessions also include information about adolescent development, teaching of emotion regulation skills such as mindfulness (for parents and adolescents) and supporting parents to reflect on their own beliefs about emotions based on their life experiences. Sessions are clearly structured, and a manual is provided to facilitators to guide sessions. Each session includes a mix of teaching, shared reflection and practicing skills. As much as possible, parents are encouraged to practice emotion coaching skills with each other using ‘live’ examples from home life and to practice at home in between sessions. Each session has a specific focus, for example, managing fear, or managing anger and conflict. Handouts are provided for each session to allow parents to review the material. A fidelity checklist for each session is used for facilitators to complete to check adherence to programme content. For further details, see TIDieR table (Additional file 2: TIDieR table for TINT).

### Training

Prior to the start of phase II RCT, participating clinicians not already trained in TINT will attend training run by the programme creators from the University of Melbourne. Regular trainings for TINT are run online and up to four clinicians from each participating team will be trained. This training is being provided for the study by the programme creators.

### Comparator: usual care in CAMHS

CAMHS are multidisciplinary teams that may include mental health nurses, social workers, family therapists, psychiatrists, and psychologists, who typically have additional training and expertise in working with young people and families. Based on the most recent national stocktake of this workforce, most staff in community CAMHS are in a clinical role (84%) and are composed of nurses (23%), social workers (18%), psychologists (16%), occupational Therapists (9%) and Psychiatrists (7%) [7]. Between 2018 and 2020/21, there was a 15% staff turnover rate nationally [7].

A case manager is typically assigned to each referred young person to coordinate care. The case manager may also be a treating clinician. Care plans are discussed and reviewed within the multidisciplinary team. Wait time to begin treatment varies depending on service capacity, with variation in wait times to receive different intervention services. For example, a young person may have to wait to start medication due to low psychiatry resource but may be able to start talking therapy sooner, or vice versa. For those with primary difficulties related to anxiety or depression, usual care may include any or a combination of case management, antidepressant medication, individual talking therapy (e.g. CBT), other therapies including group or family-based sessions, and/or liaison with the school and other agencies [7]. Data will be collected from all participants about the frequency and form of contact with CAMHS during the study period.

TINT is a structured manualised programme that will not be occurring in services as part of usual care outside of the research. However, as clinicians providing usual care may also be trained in TINT, there is potential for contamination of some content into usual care. Based on preliminary information collected from services, due to staffing and resource constraints, it is unlikely that clinicians facilitating TINT groups will also be providing a substantial amount of separate parent work. While this further reduces the likelihood of overlap of content in usual care, to assess this data will be collected on the number of total trained clinicians in a team, how many provided usual care during the study period and where possible, the amount of TINT content that may have been included in usual care sessions. Results from this will form part of the feasibility outcomes.

### Feasibility outcomes

Recruitment and retention feasibility outcomes include the following:

• Service participation: assessed by the number of services involved across the region.
• Recruitment rates: assessed as the numbers of participants expressing interest in the study, number of eligible participants and number who consent to participate.
• Retention rates: assessed by the number of participants who consent and:
- ⚬ Complete the first session of either TINT or usual care
- ⚬ Remain in the study till the first follow-up point (T1)
- ⚬ Remain in the study till the final follow-up point (T2)

Acceptability and feasibility of the intervention include the following:

• Clinician adherence to TINT: assessed by session fidelity checklists (provided in the TINT manual).
• Participant engagement: assessed by attendance records at group sessions.
• Participant and clinician experience: assessed by post-programme questionnaires, including barriers to attendance and learning.
• Depending on the results of other feasibility outcomes, interviews may be conducted with participants and/or clinicians e.g. if a particular issue is identified that warrants further exploration.
• Separation of TINT content from usual care: assessed by a number of total trained clinicians in a team, how many provided usual care during the study period and estimation of TINT content that may have been included in usual care sessions

Acceptability and feasibility of outcome measures

• Acceptability of co-design measure and other included measures assessed by completion rates.

### Outcome measures

It is anticipated that at least one co-designed outcome measure will be included alongside one or more previously validated measures (described below). This co-designed measure will not be externally validated but will be intended for use with this trial and may take the form of an additional questionnaire, scale or amendment of pre-existing standardised measures. Participants will receive a gift voucher on completing outcome measures as a thank you and to promote retention. All outcomes will be administered and recorded through REDCap. The following standardised quantitative measures will be included based on previous research with TINT and this age group and proposed mechanisms of a change via changes in emotional response and regulation [4].

#### The emotions as a child scale (EAC)

To measure changes in parent’s responses to their children’s emotions, the Emotions as a Child scale will be used. The Emotions as a Child Scale (EAC) is a 45-item measure assessing parent responses to anger, sadness and anxiety, with 15 items per emotion [37]. There are parent and youth forms which are identical other than the phrasing of perspective, with the parent form asking how the parent responds to their child’s emotions; and the youth form asking how the youth perceives their parent’s responses. The EAC has five subscales: parental encouragement, parental punishing responses, parental neglect, parental matching or magnifying and parental overriding of emotion. There are no clinical cutoff scores for this measure. Previous studies have shown acceptable internal consistency, test–retest reliability and validity metrics for various versions of the EAC with both adolescent and adult samples [38–40] [4].

#### Difficulties in emotion regulation short form (DERS-SF)

To measure changes in emotional regulation for parents and children, the Difficulties in Emotion Regulation *Short Form (DERS-SF)* will be used. This is an 18-item self-report questionnaire and is a shortened form of the original 36-item version [41]. There are six subscales: nonacceptance of emotions, difficulties engaging in goal-directed behaviour when experiencing negative emotions, impulse control difficulties, lack of emotional awareness, limited access to emotion regulation scales and lack of emotional clarity [41]. Items are rated from 1 (almost never) to 5 (almost always). There are no clinical cutoffs for this scale, and higher scores indicate greater difficulties in emotion regulation. The shortened form has been chosen to minimise the burden for participants and has comparable psychometric properties to the full version in both adult and adolescent samples [42]. Parents and 10–14-year-olds will be given the same self-report form and asked to respond about their own emotional regulation.

#### The revised child anxiety and depression scale 25 (RCADS-25)

To measure changes in child internalising symptoms, The Revised Child Anxiety and Depression Scale 25 (RCADS-25) will be used. This a questionnaire with self- and parent-report forms assessing symptoms of anxiety and depression [43]. The parent form asks about their view of their child’s whilst the child self-report form asks the child about their own experiences. In its original form, the RCADS contains 47 questions and can be interpreted using its composite subscales which assess different types of anxiety and depressive disorders. As the objective of the current study is to look at overall anxiety and depression symptoms and to minimise demands on participants, the shorter form RCADS-25 will be used. This provides an overall depression score, an overall anxiety score and a total internalizing score. In interpreting results, meaningful improvement has been suggested as at least a 50% reduction in the raw score or at least a 25% reduction for youth with treatment-resistant depression or remission defined as a T-score under 65 [44, 45]. The RCADS-25 has good psychometric properties for both overall anxiety and depression score [46, 47].

### Other data

To provide further information about the impact of TINT, a pre- and post-programme questionnaire will be completed by parents adapted from the programme developers. This will include set questions e.g. ‘How would you rate your ability to respond effectively to your child’s emotions?’ as well as space for open-ended feedback.

To assess usual care in CAMHS, all participants will be given a checklist to complete to identify the type and quantity services they received from CAMHS during the study period e.g. individual therapy, crisis intervention and medication.

Demographic information will be collected from participants including age, ethnicity and socio-economic status. Participant addresses will be collected to calculate the New Zealand Index of Deprivation as an estimate of socioeconomic status [48]. Guidance from Statistics New Zealand will be used for questions on ethnicity, gender and education.

### Data analysis

Analysis for the feasibility trial will be largely descriptive. Variables related to recruitment and retention, acceptability and feasibility of the intervention and acceptability and feasibility of the outcome measures will be described. This will include appropriate summary statistics (e.g., percentages reporting aspects as acceptable).

The statistical analyses for preliminary effect sizes from outcome measures will depend on the final measures chosen but will likely follow common methods for continuous outcomes (e.g. random-effects models). A formal statistical analysis plan will be produced prior to the start of data analysis. Analyses of trial outcomes will be based on intention-to-treat principles. The analysis will include interim measurements to allow the inclusion of participants with partial follow-up. Methods allowing for more complex handling of missing data will not be feasible within this initial study. A summary of any participant and clinician feedback will be reported.

### Progression criteria

The primary progression criterion for a fuller trial is the recruitment rate per quarter (as an absolute count). The current projection for a fuller trial suggests a sample size of at least 140 participants per arm (280 in total). The feasibility recruitment rate per quarter would need to be at least *n*=35 over 2 years of recruitment across study sites. Falling substantially short of this criterion would suggest that a subsequent study would need to recruit from a larger number of sites, potentially including a multi-region study and/or by recruiting across a longer period. This could also include expanding the team to include Māori and Pacific researchers to increase engagement with these groups as a previous trial in this area had had difficulty recruiting representative numbers from different ethnic groups [49].

### Data monitoring

The primary investigator will monitor outcome measure data. Any concerns or adverse events will be reported to the research team and responded to according to the need for example, communicating back to the treating team.

### Protocol amendments

It is anticipated that the co-design phase will lead to the amendment of outcome measures. There may also be amendments in response to feasibility findings as the trial progresses for instance related to recruitment. Any substantial amendments will be submitted to the overseeing ethics authority (Health and Disability Ethics Committee New Zealand).

## Discussion

The transition to adolescence comes with a sharp increase in the risk of developing a common mental health condition such as depression or anxiety [2]. With the prevalence of these conditions increasing over time in New Zealand as elsewhere, public mental health services for children and adolescents face increasing demand [7]. Parents are an important and under-utilised resource in treating common mental health difficulties in young adolescents. This is partly due to a lack of established evidence-based interventions. TINT is a possible addition to improve CAMHS treatment. This study will investigate the feasibility of a randomised control trial of TINT in CAMHS. Including service users in how we measure the impact of this intervention will maximise the relevance of an evaluation in this real-world setting.

### Trial status

Recruitment is currently ongoing and estimated through to end of 2022/early 2023.

## Supplementary Information

**Additional file 1.** 

**Additional file 2.**

## Data Availability

De-identified data collected in the study will be submitted to an online data repository.

## Abbreviations

TINT: Tuning in to Teens ^TM^
CAMHS: Child and Adolescent Mental Health Services
DHB: District Health Board (Organisations responsible for provision of public health and disability services in New Zealand)
CBT: Cognitive-behaviour therapy
EAC: Emotions as a Child Scale
DERS-SF: Difficulties in Emotion Regulation Scale, Short Form
RCADS-25: The Revised Child Anxiety and Depression Scale-25 item version
