# Supplementary material for: Evaluating an emotion coaching programme for parents of young adolescents attending Child Adolescent Mental Health Services (CAMHS) in New Zealand: protocol for a multi-site feasibility trial including co-design with service users
Source: Pilot Feasibility Stud. 2023 Apr 27;9:70. doi: 10.1186/s40814-023-01282-6 (PMC10134551; doi:10.1186/s40814-023-01282-6)
Supplement: Supplementary file 2 — Additional file 2. [file 40814_2023_1282_MOESM2_ESM.docx]

Template for intervention description and replication (TIDieR) table for Tuning in to Teens (TINT)^1^

| TiDieR Item | Description |
| --- | --- |
| **BRIEF NAME** |  |
| Provide the name or a phrase that describes the intervention. | TINT – Tuning in to Teens |
| **WHY** |  |
| Describe any rationale, theory, or goal of the elements essential to the intervention. | TINT is a program for parents based on emotion-socialisation theory and designed to teach parents skills to coach their children through emotional experiences. The proposed theory of change is that by increasing parental emotional regulation skills, they can better respond to their children’s emotional needs and improve child emotion regulation in turn. Specific components of emotional regulation taught include emotion awareness, emotion acceptance and meta-emotion i.e. parent’s own beliefs about emotional experiences. |
| **WHAT** |  |
| Materials: Describe any physical or informational materials used in the intervention, including those provided to participants or used in intervention delivery or in training of intervention providers. Provide information on where the materials can be accessed (e.g. online appendix, URL). | Facilitator resources:  TINT facilitator manual (provided as part of TINT training).  USB with video clips of examples of parent responses to emotions.  Eight session guideline for sessions.  Fidelity checklist for sessions.  Participant resources:  Handouts (provided with manual and on USB as part of training).  Template for emotion coaching diary to use at home.  General group resources:  Tea, coffee and snacks.  Whiteboard.  Projector or other media for viewing film clips. |
| Procedures: Describe each of the procedures, activities, and/or processes used in the intervention, including any enabling or support activities. | Groups will take place in large meeting rooms at CAMHS or other community based rooms large enough to comfortably accommodate 8-16 people. Typically, seating is set up in a circle for general group teaching and discussion. Some activities may be in pairs. ‘Fish bowl’ roleplays form a key part of practical exercises. These take the form of group roleplay where those playing the role of the young person in a parenting scenario are in the middle of the circle and everyone around acts as the ‘parent’ together. Facilitators coach and support this role plays, stepping in and demonstrating as necessary.  Each session is structured and follows manual content. The first few sessions are focused on building foundation emotion coaching skills. The last sessions have a particular theme e.g. sadness.  Eight session overview:  Session 1: The foundations for Emotion Coaching teens  Session 2: Connecting and emotional acceptance  Session 3: Building connection and showing empathy  Session 4: Self-care and Emotion Coaching sadness  Session 5: Emotion coaching fear  Session 6: Emotion coaching anger  Session 7: Responding to rejection and managing conflict  Session 8: Emotion Coaching: now and in the future  General session structure includes a warm-up, review of home practice, fish bowl in session practice, psychoeducation and discussion. Participants are encouraged to practice emotion coaching skills from the first session and additional optional home activities are also offered in each session.  Support and safety: Usual procedures in services are followed by clinicians. This includes monitoring participant wellbeing, individual check ins and follow up as needed and following standard policy. Participants may also be contacted between group sessions as needed or if requiring additional support. |
| **WHO PROVIDED** |  |
| For each category of intervention provider (e.g. psychologist, nursing assistant), describe their expertise, background and any specific training given. | Groups will be facilitated by a minimum of two clinicians trained in the intervention and an additional third to support facilitators who may or may not be trained in the intervention.  Training in TINT delivery involves attending a two day workshop (this can be in person or via zoom) provided by the program developers.  All facilitators will be current staff in local services. This means that will hold a professional health registration. Disciplines in services include Clinical Psychologists, Social Workers, Occupational Therapists and Nurses. |
| **HOW** |  |
| Describe the modes of delivery (e.g. face-to-face or by some other mechanism, such as internet or telephone) of the intervention and whether it was provided individually or in a group. | All groups are planned face to face with provision that this may be changed to video conferencing software if restrictions are in place around in person groups due to Covid-19 pandemic. |
| **WHERE** |  |
| Describe the type(s) of location(s) where the intervention occurred, including any necessary infrastructure or relevant features. | Child Adolescent Mental Health Services (CAMHS) in the Wellington region of New Zealand. These are community based outpatient public mental health services providing care for 0-18 year olds with moderate to severe mental health difficulties. |
| **WHEN and HOW MUCH** |  |
| Describe the number of times the intervention was delivered and over what period of time including the number of sessions, their schedule, and their duration, intensity or dose. | The group will be delivered in recommended format for clinical settings by program developers. This is over 8 weekly sessions of two hours long. |
| **TAILORING** |  |
| If the intervention was planned to be personalised, titrated or adapted, then describe what, why, when, and how. | The group will be extended from original 6 sessions to 8 as recommended in clinical settings.  Processes to recognise our commitment to bicultural practice for Māori in New Zealand will be included in all groups. These have been suggested based on cultural and clinical expertise and include additional welcoming process as appropriate, e.g. using a Māori karakia (prayer or incantantion) to open sessions and, use of Māori language terms. There will be no changes to manualised content. |
| **MODIFICATIONS** |  |
| If the intervention was modified during the course of the study, describe the changes (what, why, when, and how). | N/A |
| **HOW WELL** |  |
| Planned: If intervention adherence or fidelity was assessed, describe how and by whom, and if any strategies were used to maintain or improve fidelity, describe them. | Fidelity checklists that are a standard part of the TINT manual will be completed by facilitators for each session.  Group supervision will be held fortnightly and facilitated by one of the program developers and/or the lead researcher who is trained and experienced in TINT. |
| Actual: If intervention adherence or fidelity was assessed, describe the extent to which the intervention was delivered as planned. | N/A |

1. Hoffmann T, Glasziou P, Boutron I, Milne R, Perera R, Moher D, Altman D, Barbour V, Macdonald H, Johnston M, Lamb S, Dixon-Woods M, McCulloch P, Wyatt J, Chan A, Michie S. Better reporting of interventions: template for intervention description and replication (TIDieR) checklist and guide. BMJ. 2014;348:g1687.
